# Dilated Cardiomyopathy Phenotype With Global (Four‐Chamber) Involvement in a Cat: Echocardiographic, Pathological, Histopathological, and Immunohistochemical Findings

**DOI:** 10.1155/crve/9572640

**Published:** 2026-05-07

**Authors:** Giovanni Romito, Alessandra Costa, Maria Morini

**Affiliations:** ^1^ Department of Veterinary Medical Sciences, Alma Mater Studiorum–University of Bologna, Ozzano dell′Emilia, Italy

**Keywords:** cardiac histopathology, congestive heart failure, echocardiography, feline, myocardial failure

## Abstract

An 8‐year‐old castrated male Exotic Shorthair cat was referred due to the onset of abdominal effusion. Echocardiography revealed a dilated cardiomyopathy phenotype affecting both ventricles, associated with biatrial dilatation, left atrial dysfunction with spontaneous echocontrast, and right‐sided congestive heart failure. Additional diagnostic tests (thoracic radiographs, complete blood cell count, serum biochemistry, assessment of thyroid status, cardiac troponin I and plasma taurine concentrations, and urinalysis) ruled out secondary conditions able to cause a similar echocardiographic phenotype. Despite prompt initiation of cardiac therapy (furosemide, pimobendan, clopidogrel, and rivaroxaban) and subsequent hospitalization for further medical support, the cat′s clinical condition deteriorated, ultimately leading to euthanasia. Postmortem examination revealed extensive areas of myofiber thinning with a wavy appearance in both ventricles, closely resembling the attenuated wavy fiber pattern described in dogs affected by dilated cardiomyopathy. Additionally, multifocal replacement of myofibers by adipose tissue was found in both atria. Immunohistochemical analysis showed abnormal expression patterns of desmin, vimentin, and connexin 43, similar to those previously reported in humans and dogs with dilated cardiomyopathy. Given the thorough antemortem and postmortem investigations and the associated findings, a rare case of feline dilated cardiomyopathy with biventricular and biatrial involvement was diagnosed.

## 1. Introduction

In human and veterinary medicine, the dilated cardiomyopathy (DCM) phenotype is primarily characterized echocardiographically by left ventricular (LV) systolic dysfunction, with LV wall thickness often remaining within normal limits, typically progressing to LV and left atrial (LA) dilation [1–3]. In dogs, this phenotype is relatively common and may be primary or secondary to various conditions, including metabolic (hypothyroidism; hypoadrenocorticism), nutritional (taurine deficiency; grain‐free diets), toxic (doxorubicin‐induced cardiotoxicity), inflammatory (myocarditis), or tachycardia‐induced (namely, tachycardiomyopathy) [3–5]. In this species, the primary form has been associated with several genetic abnormalities (e.g., mutation involving the pyruvate dehydrogenase kinase 4 gene and striatin gene in Doberman Pinschers and Boxers, respectively) and exhibits two main histopathological patterns: the attenuated wavy fiber type and the fatty infiltration–degenerative type [6, 7].

In contrast, diagnosis of a DCM phenotype in cats is uncommon [8]. Specifically, the only form well documented in this species is that secondary to dietary taurine deficiency—first described in 1987 and now extremely rare due to taurine supplementation in commercial feline diets [8, 9]—whereas the description of other secondary forms (e.g., those due to myocarditis, sustained tachyarrhythmias leading to tachycardiomyopathy, or metabolic disorders) is mainly limited to sparse case reports [8–14]. Data on primary DCM in cats are even rarer, as the cases reported previously are predominantly based on a presumptive clinical diagnosis, often relying only on echocardiographic findings [15, 16]. As a matter of fact, to date, cases of nondiet‐related DCM in which the clinical suspicion has been confirmed by a comprehensive postmortem examination are limited to a case report in a 10‐week‐old kitten [17].

Therefore, this report is aimed at providing additional information on this still underdescribed but clinically relevant topic, by presenting for the first time in veterinary medicine a comprehensive set of clinical, cardiological, laboratory, pathological, histological, and immunohistological findings in an adult cat exhibiting a DCM phenotype of suspected primary origin.

## 2. Case Presentation

An 8‐year‐old castrated male Exotic Shorthair cat with a history of diet‐managed inflammatory bowel disease was referred for cardiologic evaluation due to the onset of an apparently unexplained abdominal effusion. This finding was identified via abdominal ultrasound performed by the referring veterinarian during routine monitoring of the extracardiac disorder. The patient was an indoor cat that was being fed a high‐quality, balanced, grain‐inclusive, gastrointestinal commercial diet^1^ and was up to date with vaccinations. Apart from the chronic intestinal disorder, no other problems were present in the past medical history.

**Figure 1:**
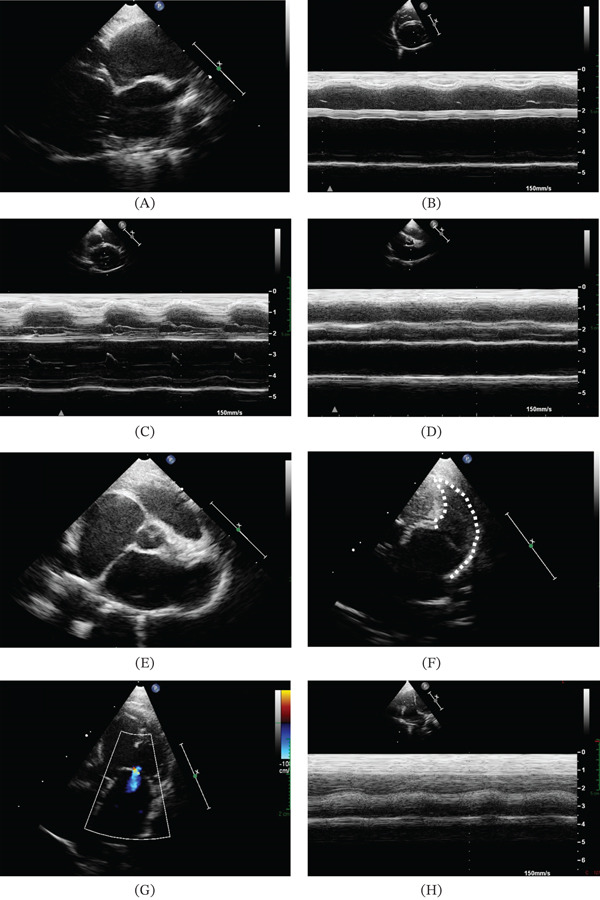
(A) Two‐dimensional right parasternal long‐axis view optimized for visualization of both atria. Note the biatrial dilation. (B, C) M‐mode echocardiographic images obtained from a right parasternal short‐axis view at the levels of the papillary muscles and mitral valve, respectively. Note the biventricular dilation and hypokinesia, more evident in the left ventricle, as demonstrated by the significantly reduced excursion of the left ventricular walls during systole and an increased mitral valve E‐point‐to‐septal separation. (D) Two‐dimensional right parasternal short‐axis view at the level of the aortic root. Note the increased left atrium to aortic root ratio. (E) M‐mode echocardiographic findings obtained from a right parasternal short‐axis view at the aortic root level. Note the reduced left atrial fractional shortening. (F) Two‐dimensional left parasternal view optimized for visualization of the left auricle. Note the dilation of this chamber (highlighted by white dotted lines) and the presence of spontaneous echocontrast. (G) Two‐dimensional left parasternal five‐chamber view optimized to visualize mild, centrally directed functional mitral regurgitation. (H) M‐mode echocardiographic findings obtained from a left parasternal view optimized for visualization of the right heart. Note the reduced tricuspid annular plane systolic excursion.

On referral, physical examination revealed a body condition score of 3/9, primarily attributed to the chronic intestinal disease, with a stable body weight of 2.5 kg compared with previous evaluations. Cardiac auscultation revealed an irregular rhythm due to the presence of some premature beats, with a mean heart rate of 200 beats/min. The remaining clinical parameters were unremarkable. Complete transthoracic echocardiography was subsequently performed according to standard techniques [18], with the cat gently restrained in right and left recumbency without the need for sedation. The examination was performed by a board‐certified cardiologist, using an ultrasound unit equipped with phased‐array transducers of various frequencies and continuous electrocardiographic monitoring.^2^ Echocardiography documented a DCM phenotype involving both ventricles, associated with mild, centrally directed mitral and tricuspid regurgitation of suspected functional origin (given the ventricular enlargement and absence of evident structural valvular abnormalities), biatrial dilation, LA dysfunction with spontaneous echocontrast, and ultrasonographic evidence of right‐sided congestive heart failure (caudal vena cava and hepatic vein congestion with mild abdominal effusion) (Table 1; Figure 1; Video S1–S2). Isolated ventricular premature complexes were documented electrocardiographically.^3^ Additional tests included thoracic radiographs, a complete blood cell count, serum biochemistry, assessment of thyroid status and concentration of serum cardiac troponin I and plasma taurine, and urinalysis. Thoracic radiographs revealed only an increased cardiac silhouette size (Vertebral Heart Scale: 9.5) [19]. Complete blood count, serum biochemistry, concentration of thyroid hormone, cardiac troponin I and plasma taurine, and urinalysis were overall unremarkable. Cardiac treatment was prescribed, including oral furosemide (1 mg/kg q12h), pimobendan (0.25 mg/kg q12h), clopidogrel (18.75 mg/cat q24h), and rivaroxaban (2.5 mg/cat q24h) [20]. Daily monitoring of resting respiratory rate and scheduled rechecks were recommended to the owners, with the first follow‐up planned within 1 week.

**Table 1:** Selected echocardiographic findings.

Selected echocardiographic Parameters	Values^#^	Reference limits	References
Left‐sided cardiac chambers			
IVSd (mm)	3.5	2.6–4.5∗	a
LVFWd (mm)	3.4	2.5–4.4∗	a
LVDd (mm)	19.5	10.9–17.0∗	a
LVDs (mm)	17.5	4.8–11.2∗	a
LVFS (%)	10%	28–62∗	a
EPSS (mm)	6	1.2 ± 0.5	b
LA/Ao	1.8	0.86–1.41∗	a
LAD (mm)	18	< 16	c
LAFS (%)	8	> 25	c
Vmax‐Lau (cm/s)	16	> 25	d
Right‐sided cardiac chambers			
RAD (mm)	18.5	10.1–12.3	e
RVDd (mm)	13.5	6.7 ± 1.4	e
RVDs (mm)	9.5	3.4 ± 1.1	e
TAPSE (mm)	4	9.1 ± 1.4	e
Vmax‐RV S′ (cm/s)	4	—	—

*Note:* Number sign (^#^) denotes that the values reported in the table are the result of the mean of measurements performed over three consecutive cardiac cycles during phases of sinus rhythm. Asterisk (∗) denotes body weight–based reference limits (2.5 kg cats).

Abbreviations: EPSS, mitral valve E‐point‐to‐septal separation; IVSds, end‐diastolic interventricular septum thickness; LA:Ao ratio, left atrium to aortic root ratio; LAD, left atrial anteroposterior diameter; LAFS, left atrial fractional shortening; LVDd, left ventricular end‐diastolic diameter; LVDs, left ventricular end‐systolic diameter; LVFS, left ventricular fractional shortening; LVFWd, end‐diastolic left ventricular wall thickness; RAD, right atrial anteroposterior diameter; RVDd, right ventricular end‐diastolic diameter; RVDs, right ventricular end‐systolic diameter; TAPSE, tricuspid annular plane systolic excursion; Vmax‐Lau, peak velocity of the left atrial auricle blood flow; Vmax‐RV S′, tissue Doppler imaging‐derived peak of the systolic longitudinal myocardial motion velocity recorded at the lateral tricuspid annulus.

^a^J. Häggström, Å. O. Andersson, T. Falk, et al., “Effect of Body Weight on Echocardiographic Measurements in 19,866 Pure‐Bred Cats With or Without Heart Disease”, Journal of Veterinary Internal Medicine, 30 (2016), 1601–1611, 10.1111/jvim.14569.

^b^A. Brahmwar, P. Hase, R. Gaikwad, et al., “M‐Mode Echocardiographic Studies in Healthy Cats”, Indian Journal of Veterinary Sciences and Biotechnology, 20 (2024), 120–123, 10.48165/ijvsbt.20.3.24.

^c^A. Machado, C. Partington, J. Silva, et al., “Left Atrial Fractional Shortening in Cats: A Comparison Between Two Echocardiographic Views”, Journal of Veterinary Cardiology 55 (2024), 38–47, 10.1016/j.jvc.2024.08.002.

^d^K. E. Schober, I. Maerz, “Doppler Echocardiographic Assessment of Left Atrial Appendage Flow Velocities in Normal Cats”, Journal of Veterinary Cardiology, 7 (2005), 15–25, 10.1016/j.jvc.2004.11.001.

^e^L. C. Visser, C. Q. Sloan, J. A. Stern, “Echocardiographic Assessment of Right Ventricular Size and Function in Cats With Hypertrophic Cardiomyopathy”, Journal of Veterinary Internal Medicine 31 (2017), 668–677, 10.1111/jvim.14688

Regrettably, over the following days, the cat′s clinical condition progressively deteriorated at home, with decreased appetite and the onset of tachypnea. Consequently, 4 days after the initiation of cardiac therapy, the cat was returned to the hospital. On presentation, physical and diagnostic findings were consistent with bilateral congestive heart failure (abdominal effusion and diffuse pulmonary B‐lines) and cardiogenic shock (systolic arterial blood pressure measured using a high‐definition oscillometric device:^4^ 86 mmHg; heart rate: 140 beats/min; rectal temperature: 32°C). The cat was hospitalized in the intensive care unit under continuous electrocardiographic monitoring, which demonstrated sinus rhythm intermittently interrupted by rare, isolated ventricular premature complexes. Intravenous furosemide (1 mg/kg at arrival, followed by five additional boluses at the same dose over the first day) and dobutamine (constant rate infusion 5 *μ*g/kg/min) were promptly administered. Additionally, a nasal cannula for oxygen supplementation was placed. Moreover, pimobendan, clopidogrel, and rivaroxaban were continued at the same dosages. Despite intensive care, the cat′s condition continued to worsen. The owner elected euthanasia on the second day of hospitalization.

A complete necropsy was performed. Moderate serous pleural and peritoneal effusions, consistent with transudate, were observed. Within the thoracic cavity, opening of the pericardium revealed a globoid, diffusely dilated heart. The lungs were enlarged and had a dark red parenchyma. Upon sectioning, a large amount of frothy fluid exuded from the parenchyma and lower airways. No macroscopically significant lesions were present in any of the other organs. The entire heart, along with representative samples from the lung, liver, kidney, pancreas, small and large intestine, and mesenteric lymph node, was immediately fixed in 10% buffered formalin. Following 48 h of formalin fixation, the heart was removed for further macroscopic examination and trimming. A base‐to‐apex–oriented cross‐section of the heart revealed moderate to marked biatrial dilatation with a rounded profile and thinning of the walls of both ventricles. Multiple myocardial tissue specimens were collected from both atria, the ventricular free walls of both ventricles, and the interventricular septum, then routinely processed for histology and stained with hematoxylin and eosin. Additional histochemical stains, including Masson′s trichrome and phosphotungstic acid hematoxylin, were performed to highlight connective tissue, as well as cross‐striations and intercalated discs of myocardial fibers, respectively. Immunohistochemical analyses (desmin [DES], vimentin, and connexin 43 [Cx43]^5, 6, 7, 8^), using the avidin–biotin–peroxidase method, were also performed on representative samples of the heart.

**Figure 2:**
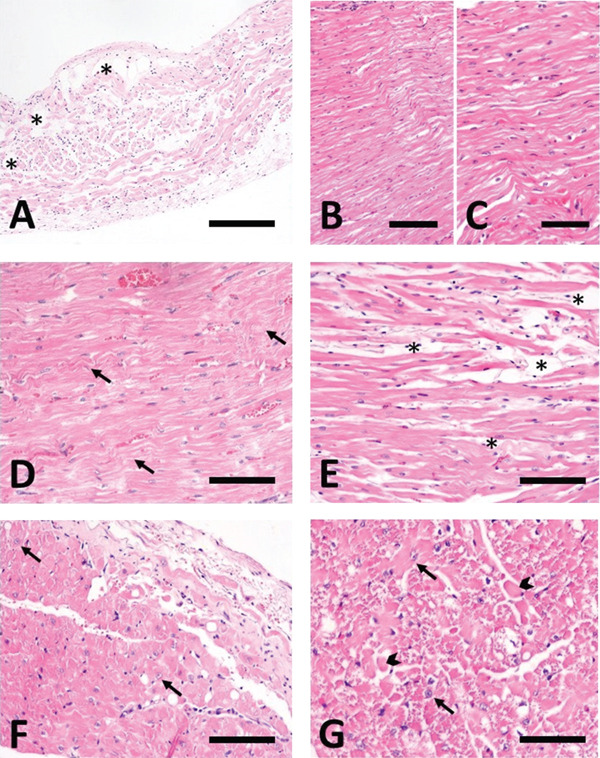
Histological findings. (A) Section of the right atrial myocardium. Multifocal fatty infiltration (asterisks) of the atrial wall surrounded by hypotrophic cardiomyocytes. Hematoxylin and eosin stain. Bar: 500 *μ*m. (B, C) Left and right ventricular myocardium, respectively. Myofibers are thin with a wavy appearance. Hematoxylin and eosin stain. Bar: 400 and 300 *μ*m, respectively. (D) Left ventricle. Note that the myofibers are thinner than normal and show a wavy arrangement (arrows). Hematoxylin and eosin stain. Bar: 300 *μ*m. (E) Right ventricle. Note the atrophied myofibers, which appear separated by clear spaces indicative of edematous fluid (asterisks). Hematoxylin and eosin stain. Bar: 350 *μ*m. (F, G) Signs of cardiomyocyte degeneration observed in the intraventricular septum. Note the histomorphologic changes in cardiomyocytes, including cytoplasmic vacuolization, fragmentation, and altered staining (arrowheads), along with scattered abnormal cell nuclei (arrows). Hematoxylin and eosin stain. Bars: 350 *μ*m. IVS, interventricular ventricular septum; RA, right atrium; LV, left ventricle; RV, right ventricle.

**Figure 3:**
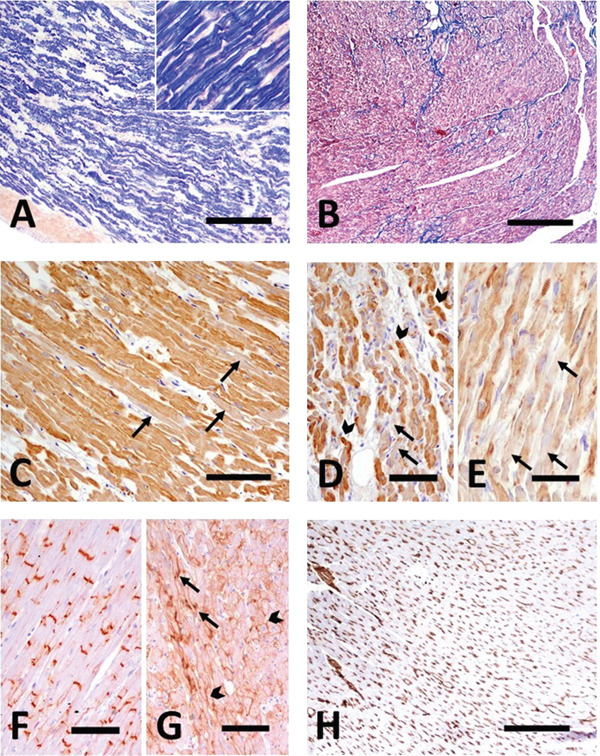
Special histochemical and immunohistochemical stains from samples of the left and right ventricular myocardium. (A) Histochemical stain with hematoxylin–phosphotungstic acid highlights the disorganization and thinning of myofibers, as well as their wavy appearance. Inset: higher magnification detail. Bar: 500 and 50 *μ*m, respectively. (B) Cords of collagen (blue staining) are evident. Masson′s trichrome stain. Bar: 500 *μ*m. (C–E) Cytoplasmic expression of desmin in cardiomyocytes (brown staining). The cardiomyocytes are swollen and show irregular and weak desmin expression (C, arrows) or are free of desmin (D and E, arrows), with no visible cross‐striation. Overcolored aggregates of desmin are also visible (D, arrowheads). Hematoxylin counterstain. Bar: 300 (C, D) and 100 *μ*m (E). (F, G) Connexin 43 expression in control myocardial tissue from a healthy cat and the cat from the present report, respectively. In the affected cat, connexin 43 shows alterations in positivity (solid bands in contact zones), presented as granules in the cytoplasm and accumulation in the outer membrane periphery of the cardiomyocytes (lateralization). Hematoxylin counterstain. Bar: 300 *μ*m. (H) Note the abundant vimentin‐positive cells between cardiomyocytes, which is evidenced by the cytoplasmic expression (brown staining) in interstitial tissue. Hematoxylin counterstain. Bar: 500 *μ*m.

Histological examination of the heart revealed multifocal areas of myofiber thinning (< 6 *μ*m in diameter) with a wavy appearance in the free walls of both ventricles and the interventricular septum, involving approximately half of the myocardial thickness overall. Moreover, multifocal clusters of contracted, hypereosinophilic myofibers displaying loss of striations, hyalinised sarcoplasm, and pyknotic nuclei—consistent with myofiber degeneration—were also observed in the LV myocardium. Focal replacement of myofibers by adipose tissue was noted in both atria (Figure 2). Additional findings included a slight increase in interstitial fibrous tissue, characterized by thin connective tissue septa that stained blue with Masson′s trichrome (Figure 3B).

Immunohistochemical analysis showed unusual expression patterns for both DES and Cx43, including areas with absent or reduced DES expression, occasional granular staining (DES and Cx43), and lateralization of Cx43 immunoreactivity (Figure 3C–G). Vimentin disclosed a high number of immunoreactive cells between cardiomyocytes (Figure 3H). No inflammatory infiltrates or other myocardial or valvular alterations were observed. Extracardiac findings included severe pulmonary capillary congestion associated with diffuse, pale eosinophilic alveolar transudate and a moderate number of hemosiderin‐laden macrophages. Additionally, there was mild, chronic lymphoplasmacytic enteritis.

## 3. Discussion

In cats, DCM is among the rarest acquired heart diseases, accounting for less than 5% of cases of feline cardiomyopathies [8]. In addition to primary DCM, differential diagnoses for a DCM phenotype in this species currently include taurine deficiency, atypical presentation of other cardiomyopathies (end‐stage hypertrophic cardiomyopathy; arrhythmogenic cardiomyopathy with biventricular involvement), myocarditis, sustained tachyarrhythmias leading to tachycardiomyopathy, metabolic disorders (advanced hyperthyroidism; severe hypocalcemia), and rare genetic/familial disorders (endocardial fibroelastosis) [8–14]. Although the list of differential diagnoses reported above might suggest that the scientific literature on echocardiographic DCM phenotypes in cats is extensive, it is important to consider that, apart from the nutritional form, the description of other secondary forms is largely based on sporadic case reports [8–14]. Moreover, the data available to date on primary DCM in cats are mostly limited to publications in which the clinical suspicion was based solely on echocardiographic findings [15, 16].

Accordingly, the present case appears to differ from previous feline literature, as a multimodal investigation was conducted both antemortem and postmortem, as performed in human [21, 22] and canine cardiology when managing myocardial diseases [23]. Thanks to this systematic approach, the findings obtained allowed the exclusion of the aforementioned differential diagnoses, supporting the hypothesis of a primary form of DCM. Specifically, the unremarkable findings on laboratory tests (complete blood count, serum biochemistry, and assessment of the concentration of thyroid hormone, cardiac troponin I, and plasma taurine) ruled out metabolic conditions capable of impairing LV systolic function. Moreover, the cardiological evaluation allowed the exclusion of sustained tachyarrhythmias, making tachycardiomyopathy unlikely. Additionally, postmortem examination revealed no signs indicative of hypertrophic or arrhythmogenic cardiomyopathy or myocarditis, making it unlikely that LV systolic dysfunction was the result of an atypical presentation of any of these conditions. Finally, a histological picture of endocardial fibroelastosis was ruled out [8–14].

Unlike in humans and dogs—two species in which various genetic abnormalities have been shown to contribute to the pathogenesis of primary DCM [24]—causative mutations have not yet been identified in feline DCM (although a genetic contribution has been suggested in cats as well [25]). Accordingly, in contrast to humans and dogs [6], the clinical suspicion of a primary DCM phenotype in cats cannot currently be supported antemortem by genetic testing and may only be further confirmed through postmortem evaluation [8], as done in the present report.

As previously stated, a thorough postmortem evaluation of the present case identified several findings critical for diagnostic characterization. However, it should be considered that, due to the paucity of data on pathological alterations in feline DCM, the interpretation of our findings—particularly the histopathological and immunohistochemical ones—relied in part on comparison with previously documented observations in human and canine DCM. In the cat from this report, the predominant LV histomorphologic features were consistent with those described in many dogs with primary DCM, specifically the attenuated wavy fiber type [7]. This pathological pattern represents the main explanation of the LV systolic dysfunction observed echocardiographically. Interestingly, in the case here reported, the same pattern was documented not only in the LV free wall and interventricular septum but also in the right ventricle, explaining the concomitant right ventricular (RV) systolic dysfunction of our cat. In humans, primary DCM does not exclusively affect the left ventricle. Indeed, RV dysfunction can occur in 20%–65% of cases, not only as a result of left‐sided heart disease (e.g., type 2 pulmonary hypertension or RV hypoperfusion caused by LV failure), but also due to a primary process affecting the RV cardiomyocytes [26, 27]. In contrast, the right ventricle has been largely overlooked in feline primary DCM, with only one prior report documenting its echocardiographic and pathological involvement in a kitten with a DCM phenotype of unknown origin [17]. Beyond RV abnormalities, this report also highlights concomitant LA and right atrial involvement, with evidence of areas of adipose myofiber replacement. These findings likely explain the LA dysfunction identified on echocardiography. Pathological changes affecting both atria have been documented in human DCM [26, 28]. Moreover, LA structural abnormalities have been described in dogs with DCM, including fatty infiltration [29], as documented in the present case. Conversely, atrial pathology in feline DCM remains poorly documented to date. Therefore, our histological findings appear to be novel and add to the limited body of literature on this topic, indicating that feline DCM may involve not only the left ventricle but also other cardiac chambers, as reported in humans and dogs.

A final consideration concerns the immunohistochemical findings related to DES (an intermediate filament protein involved in cytoskeletal organization and maintenance of cardiomyocyte structural integrity), Cx43 (a gap junction protein expressed in the heart, involved in electrical conduction and coordinated contraction), and vimentin (an intermediate filament protein primarily produced by fibroblasts, endothelial cells, and smooth muscle cells, involved in cellular migration, adhesion, and division), proteins which play an essential structural, mechanical, and regulatory role in cardiac integrity [30–34]. Although comparison of our data with findings from other studies on feline DCM is limited by the lack of prior investigations on immunohistochemistry in cats with this cardiomyopathy, it is noteworthy that our results closely align with those reported in humans and dogs. Indeed, in these species, it has been demonstrated that DCM can be associated with decreased expression of DES and Cx43, along with an increased number of interstitial vimentin‐positive cells—abnormalities that may serve as a substrate predisposing affected subjects to ventricular dysfunction and arrhythmias [30–34]. Therefore, our findings may be useful to provide additional data to further deepen the comparison among human, canine, and feline DCM.

Data from the present case report should be interpreted in light of possible limitations, such as the lack of speckle‐tracking echocardiography to provide a more detailed assessment of LV and LA function [35, 36], and immunohistochemical analyses limited to the investigation of DES, vimentin, and Cx43.

## Funding

Open access publishing was facilitated by Universita di Bologna, as part of the Wiley–CRUI‐CARE agreement.

## Conflicts of Interest

The authors declare no conflicts of interest.

## Endnotes

^1^Gastrointestinal Biome, Hill′S Pet Nutrition Italia S.R.L., Roma, Italy.

^2^iE33 ultrasound system, Philips Healthcare, Monza, Italy.

^3^Cube ECG, Cardioline S.p.A., Caverano, Italy.

^4^petMAP graphic, Ramsey Medical Inc., Tampa, United States.

^5^Desmin (RD301), 1:250, Santa Cruz Biotechnology, Texas, United States.

^6^Vimentin (V9), 1:600, Santa Cruz Biotechnology, Texas, United States.

^7^ Connexin 43 (F‐7), 1:250, Santa Cruz Biotechnology, Texas, United States.

^8^ Vectastain, Vector Laboratories, Burlingame, United States.

## Supporting Information

Additional supporting information can be found online in the Supporting Information section.

## Supporting information


**Supporting Information 1** Video S1: Transthoracic echocardiographic video clip obtained from a right parasternal long‐axis four‐chamber view. The video speed has been purposefully reduced to allow accurate observation of the global (four‐chamber) dilation and dysfunction. Of note, the hyperechoic area identifiable at the level of the parietal leaflet of the tricuspid valve is not attributable to an anatomical abnormality but is primarily the result of a setting‐related artifact.

**Supporting Information 2** Video S2: Transthoracic echocardiographic video clip obtained from a right parasternal short‐axis view at the level of papillary muscles. Also in this case, the video speed has been purposefully reduced to allow accurate observation of the biventricular dilation and dysfunction.

## Data Availability

The data that support the findings of this study are available on request from the corresponding author. The data are not publicly available due to privacy or ethical restrictions.
